# Nano-insecticides against the black cutworm *Agrotis ipsilon* (Lepidoptera: Noctuidae): Toxicity, development, enzyme activity, and DNA mutagenicity

**DOI:** 10.1371/journal.pone.0254285

**Published:** 2022-02-03

**Authors:** Mona Awad, El-Desoky S. Ibrahim, Engy I. Osman, Wael H. Elmenofy, Abdel Wahab M. Mahmoud, Mohamed A. M. Atia, Moataz A. M. Moustafa

**Affiliations:** 1 Faculty of Agriculture, Department of Economic Entomology and Pesticides, Cairo University, Giza, Egypt; 2 Faculty of Agriculture, Department of Genetics, Cairo University, Giza, Egypt; 3 Agricultural Genetic Engineering Research Institute, ARC, Giza, Egypt; 4 Faculty of Agriculture, Plant Physiology Section, Botany Department, Cairo University, Giza, Egypt; 5 Molecular Genetics and Genome Mapping Laboratory, Genome Mapping Department, Agricultural Genetic Engineering Research Institute (AGERI), Agricultural Research Center (ARC), Giza, Egypt; University of Carthage, TUNISIA

## Abstract

Frequent applications of synthetic insecticides might cause environmental pollution due to the high residue. In addition, increasing insecticide resistance in many insect pests requires novel pest control methods. Nanotechnology could be a promising field of modern agriculture, and is receiving considerable attention in the development of novel nano-agrochemicals, such as nanoinsectticides and nanofertilizers. This study assessed the effects of the lethal and sublethal concentrations of chlorantraniliprole, thiocyclam, and their nano-forms on the development, reproductive activity, oxidative stress enzyme activity, and DNA changes in the black cutworm, *Agrotis ipsilon*, at the molecular level. The results revealed that *A*. *ipsilon* larvae were more susceptible to the nano-forms than the regular forms of both nano chlorine and sulfur within the chlorantraniliprole and thiocyclam insecticides, respectively, with higher toxicities than the regular forms (ca. 3.86, and ca.2.06-fold, respectively). Significant differences in biological parameters, including developmental time and reproductive activity (fecundity and hatchability percent) were also observed. Correspondingly, increases in oxidative stress enzyme activities were observed, as were mutagenic effects on the genomic DNA of *A*. *ipsilon* after application of the LC_50_ of the nano-forms of both insecticides compared to the control. These promising results could represent a crucial step toward developing efficient nanoinsecticides for sustainable control of *A*. *ipsilon*.

## Introduction

The black cutworm, *Agrotis ipsilon* (Hufnagel) (Lepidoptera: Noctuidae) is a major insect pest that can destroy many important crops worldwide [1]. Specifically, the insect larvae damage many crop species, vegetables, and weeds [2]. *A*. *ipsilon* larvae can consume over 400 cm^2^ of foliage during their development [3]. Chemical insecticides have been used to prevent crop loss by *A*. *ipsilon* [4].

The selection of highly effective insecticides and their appropriate application methods is a fundamental problem of integrated pest management strategies. Nanotechnology could provide new methods and agricultural products to counteract the fears related to the potential unwanted environmental impact of chemical insecticides through increased exposure and toxicity in non-target organisms [5]. Nanoparticle technology could develop through two distinct mechanisms: (a) supplying singular crop protection or (b) ascarriers for existing pesticides [6]. Generally, nanopesticides are defined as any pesticide formulation of nano-sized, insecticide particles or small, engineered structures with pesticide properties [7,8]. Nano-pesticides have several advantages, including increased potency and durability, and a reduced amount of active ingredients [9]. They are also considered to be a promising solution for the reduction of the environmental footprint left by chemical pesticides [7]. The increasing interest in the use of nano-pesticides raises questions about their fate, toxicity, and biodegradation [10], as well as how to assess the environmental risk of these materials.

The introduction of nano-insecticides into the environment necessitates the careful identification of their potential. Generally, insecticide studies focus on evaluating the lethal and sublethal toxicity of chemical active substances on insect development and enzyme activities [11]. Insecticides are considered a stress factor that may upset the functional balance in insects [known as oxidative stress (OS)]. Insecticides are characterized by the enhanced production of reactive oxygen species (ROS) with the simultaneous impairment of their scavenging systems. Increased ROS concentrations result in oxidative damage to proteins, lipids, and nucleic acids, and thus cell function, organs, and the entire organism may be seriously disrupted, resulting in death [12]. To avoid, or at least reduce this effect, organisms have developed effective defense systems controlled by their nervous and endocrine glands. The oxidative stress enzymes are an essential group of enzymes that minimize the adverse effects of ROS on cells. Several defense mechanisms, including enzymatic and non-enzymatic components, have been developed by insects [13,14]. Superoxide dismutase (SOD), catalase (CAT), peroxidase (POX), glutathione (L-ɣ-glutamyl-L-cysteinylglycine, GSH) are major antioxidant enzymes in insects that play a fundamental role in cell protection by removing oxidative stress [15]. SOD is an antioxidant enzyme, which converts superoxide into oxygen and hydrogen peroxide [16]. CAT is mainly a H_2_O_2_-scavenging enzyme that principally removes H_2_O_2_ generated from developmental or environmental stimuli into water and oxygen in all aerobic organisms [17]. CAT tends to reduce small peroxides, such as H_2_O_2_, but does not affect larger molecules, such as lipid hydroperoxides. POX utilizes either H_2_O_2_ or O_2_ to oxidize a wide variety of molecules [18], and uses H_2_O_2_ to oxidize phenolic compounds. GSH is the heart of the essential cellular antioxidant system [19], and may serve as an electron donor (cofactor) for antioxidant enzymes like glutathione peroxidases and glutathione S-transferases [20].

Genetic molecular markers have become a central tool to determine the degree of genetic variability [21], molecular phylogenetics [22], genetic fidelity [23], and disease resistance [24] in organisms. Various kinds of molecular marker techniques have been identified in insect populations [25]. The inter-simple sequence repeats (ISSR) are a useful marker tool to detect genetic variation and differentiate closely-related individuals [26]. The high variability level of the ISSR marker has been indicated to be a common characteristic of Lepidoptera genomes and is applied as a fingerprint technique between groups of organisms [27].

Due to the lack of information on nanoinsecticide toxicity, several studies are required to better understand their effects on the biological and physiological parameters of target insects [10]. There are currently no sufficient screening methods to assess whether nanoinsecticides are safe for field administration without significant side effects on human health. Thus, this study’s main goal was to evaluate the potential effects of the nano-forms of two new insecticides, chlorantraniliprole and thiocyclam, on *A*. *ipsilon*. We also investigated the effects of the lethal and sublethal concentrations of both insecticides and their nano-forms on the development, reproductive activity, and oxidative stress enzyme activities, including; SOD, CAT, lipid peroxidase, and GR of *A*. *ipsilon*. This study examined both insecticides for nanoparticle-induced changes in *A*. *ipsilon* at the DNA molecular level.

## Materials and methods

### Insect rearing

*Agrotis ipsilon* was reared for 12 generations in the absence of insecticides in the laboratory in a rearing room at 26 ± 1°C, 65 ± 5% relative humidity and a photoperiod of 16 L:8 D. The newly hatched larvae were kept in a clean glass jar (1 L) and provided a castor oil leaves daily until the third instar larvae emerged. Then they were transferred to larger, and clean glass jars (2 L) to prevent larval cannibalism. The bottom of each jar was covered with a thick layer of fine sawdust and the usual rearing techniques were performed along with the developing instars larvae until pupation occurred. After pupation, the developed pupae had been transferred to clean jar for the adult emergence. The adult moths (males and females in ratio 7:5) were transferred to a glass jar (5 L) which was supplied with hanged piece of cotton wool soaked in 10% sugar solution as dietary supplement [11].

### Insecticides and chemicals

Chlorantraniliprole (Coragen^®^ 20% SC, suspension concentrate, DuPont) and thiocyclam (Evisect-S^®^ 75% SC) were tested. All chemicals used in the preparation of the nano-form of the insecticides were purchased from Sigma Chemical Co. (St. Louis, USA) without further purification. The oxidative stress enzymes kits were purchased from Biodiagnostic Company, Egypt.

### Nanoinsecticides preparation and size measurements

Nano-chlorine (chlorantraniliprole) and nano-sulfur (thiocyclam) were prepared by De Oliveira et al. [28], Cota-Arriola et al. [29], and Xu et al. [30], with some modifications. Hydrochloric acid (HCl) and orthorhombic bravais were used as a chlorine source, while sodium thiosulphate and sulfuric acid were used as a sulfur source. The nano-chlorine was made from a sedimentary solution of HCl and MnO2 v:v, where hydrochloric acid and manganese oxide were added slowly in a molar ratio (3:2) in the presence of the stabilizing agent PVA using forceful moving for 5 hours continuously. The obtained precipitation was filtered and washed thoroughly with deionized water, then 16 ml of NaCl 0.2 M aquwous solution was added to the suspension solution using forceful moving at a steady rate for 40 min. The reaction fusion was stirred for an additional 3 hours continuously at ambient temperature. The precipitate was mixed with orthorhombic brava by w:w (2:1) in the presence of HCl 90%, then centrifuged at 1500 rpm for 30 min. Then, the solution was cooled in an ice bath, subsequently exposed to 1.5 psi pressure continuously for 6 hours, and the final nano-suspension was ready for examination under transmission electron microscope.

Nano-sulfur was prepared from a sedimentary solution of sodium thiosulphate and sulfuric acid (1:1). Sodium polysulfide and hydrochloric acid solutions were incrementally mixed in a molar ratio of 3:2 under forceful moving for 8 hours discontinuously. The obtained precipitation was filtered and washed methodically with deionized water in a mixed water/toluene system, then washed with ionized water for 3 hours continuously. The precipitation was mixed with oxalic acid 1 M and trimethylammoniumbromide compound solution in molar ratio 1:3 under slow stirring at 33°C for 6 hours discontinuously. Afterwards, drop benzene sulphonate in molar ratio 2:3 was added, and the obtained solution was kept at 1.5 psi pressure for 3 days discontinuously (7 hours per day). Finally, the solution was dried in an oven at 90°C for 3 days continuously. The final nano-suspension was prepared in deionized water and left on a shaker for 2 days continuously at 20°C. The final nano-suspension was ready for examination under transmission electron microscope.

### Bioassays

The toxicity of chlorantraniliprole, thiocyclam, and their nano-forms on the second instar larvae were assessed using the leaf dipping technique [31], with some modifications. Briefly, six different concentrations were prepared for each insecticide, while water was used for the control. Castor oil leaves were dipped in each concentration for 20 s then left to air-dry. After drying, the leaves were transferred into a glass jar (0.25 L). Ten larvae were added to each jar with five replications and left to feed for 24 h. Afterwards, all larvae were offered untreated leaves, and the mortality% was recorded four days (96 hours) post-treatment [32] to calculate the lethal and sublethal concentrations for each insecticide form. The bioassay was repeated twice.

### Effects on *Agrotis ipsilon* development

Lethal concentrations that kill 15 and 50% of the larvae exposed to each form of chlorantraniliprole and thiocyclam were applied on the second instar larvae using the method described above. Surviving insects were used to study the effect of each insecticide on larval and pupal development time, pupation%, and adult emergence. Seven days after exposure, the surviving larvae were transferred individually to a clean cup to record the development time of the larval and pupal stages and the pupation%. After pupation, each pupa was sexed, weighed, and kept individually in the same cup to record the emergence%.

### Studies on fecundity and fertility

Groups of 5 females and 7 males in 3 replicates [33] were used to calculate the number of eggs and hatching% after the second instar larvae were treated with the LC_15_ and LC_50_ values of each insecticide and their nano-forms. Deposited eggs were collected and counted on days 2–6 in the mating jars. The eggs were transferred to a clean jar and kept for 5 days to record the hatching%.

### Oxidative stress enzyme assays

#### Sample preparation

Seven days after LC_15_ and LC_50_ equivalent treatment of the second instar larvae, 100 mg fresh body weight of the surviving larvae were transferred to clean and sterile Eppendorf tubes (1.5 ml). The samples were stored immediately at −20°C until later analysis. Each treatment and control was replicated five times. The treated larvae were homogenized in a potassium phosphate buffer (50 mM, pH 7.0) at 30 μl buffer per 1 mg of body weight. The homogenate was centrifuged for 15 min at 7000 g at 4°C, and the supernatants were used for further analysis.

#### Enzymes measurement

SOD activity was determined according to Misra and Fridovich [34] at the absorbance of 560 nm. The CAT enzyme activity was estimated by measuring the rate of H_2_O_2_ consumption [35] via absorbance at 510 nm. The level of lipid peroxidase was assayed by monitoring the formation of malondialdehyde (MDA) at 534 nm [36]. GR activity was estimated as the reduced glutathione (GSSG) in the presence of NADPH [37], which oxidizes to NADPH^+^ at 340 nm. The total protein concentration of all samples was measured spectrophotometrically based on the Biuret Method using Protein Biuret Kit (Biodiagnostic, Egypt).

### Molecular analysis

#### DNA extraction

DNA was isolated from both treated and untreated second instar larvae using a G-spin™ total DNA extraction kit (INtRON Biotechnology) following manufacturer’s instructions. The DNA was quantified with a Qubit 4 Fluorometer (Thermo Fisher Scientific Inc.). The DNA concentrations were measured and subsequently adjusted in all samples to 10 ng/μL for subsequent molecular analyses.

#### ISSR polymorphism analysis

For ISSR PCR amplification [38,39], a set of 15 ISSR primers were applied against the 9 treatments. The PCR was carried out in a total volume of 25 μl containing the following components: 25 ng genomic DNA; 1X PCR buffer; 1.5 mM MgCl_2_; 0.25 mM of each dNTPs; 1 μM of each primer; 1 U Go-Taq Flexi polymerase (Promega).

Thermocycling amplification was performed with a GeneAmp PCR system 9700 (Applied Biosystem, Inc.). The amplification was programmed at 94°C for 5 min for the initial denaturation cycle, followed by 35 cycles with each cycle comprising 94°C for 1 min, 50°C for 1 min, then 72°C for 90 s; and a final extension at 72°C for 7 min. The produced PCR amplicons were electrophoresed using 1.5% agarose gel. A 100 bp plus DNA ladder and 1 kb were used as molecular size standards. PCR products were photographed using a Gel Doc™ XR+ System (Bio-Rad®).

### Data analyses

#### Biological and biochemical data analyses

The statistical analysis program LDP line was used to determine the lethal and sublethal concentration values (LC_15_, LC_50_, and LC_90_) for each insecticide and its nano-forms (with 95% confidence limits). All biological parameters and oxidative stress enzymes activity (SOD, CAT, lipid peroxidase, and GR) were performed using one way ANOVA in addition to Dunnett’s multiple comparisons test with Graph Pad Prism 8 statistical analysis software. Moreover, a silhouette analysis was performed to evaluate the quality of the reproductive activity, enzymes, and developmental measurements by testing the cluster distances within and between each cluster [40]. Additionally, we performed a multidimensional preference analysis to disclose the interrelationships among parameters in addition to the similarity classification in terms of dependent and independent variables in different space dimensions [41]. Finally, hierarchical clustering based on the correlation analysis was conducted with two-dimensional heatmap plotting was constructed.

#### Molecular data analysis

For ISSR data analysis, the generated amplicons were scored visually. To generate a binary data set, the amplicons were scored as absent (0) and present (1). The polymorphism percentage was analyzed by dividing the number of amplified polymorphic bands by the total number of amplified bands separately for each primer [42]. A similarity matrix was built to estimate the genetic distances between all possible treatment pairs. The Jaccard coefficient was used for the pairwise comparisons [43]. The genetic similarity (GS) between each pair of treatments was calculated using GS = *a*/(*n*-*d*), in which n is the total number of fragments; a is the number of positive coincidences; and d is the number of negative coincidences. The genetic distances (GD) between pairs of treatments were estimated using GD = 1-GS. The unweighted pair group method of arithmetic averages (UPGMA) was used to construct the dendrogram [44].

The efficiency of the ISSR primers was determined by calculating the following parameters: expected heterozygosity (H = 1 – Σ pi^2^ according to Liu [45]; polymorphism information content (PIC = 1 – Σ pi^2^ – Σ pi^2^) according to Botstein et al. [46]; effective multiplex ratio (E = n β) according to Powell et al. [47]; marker index (MI = E Hav) according to Powell et al. [47]; mean heterozygosity (Hav = Σ H_n_/n_p_) according to Powell et al. [47]; discriminating power (D = 1 – C) according to Tessier et al. [48]; resolving power (R = Σ I_b_) according to Prevost and Wilkinson [49].

## Results

### Characterization of nano-(chlorine) chlorantraniliprole and nano-(sulfur) thiocyclam

**Fig 1:**
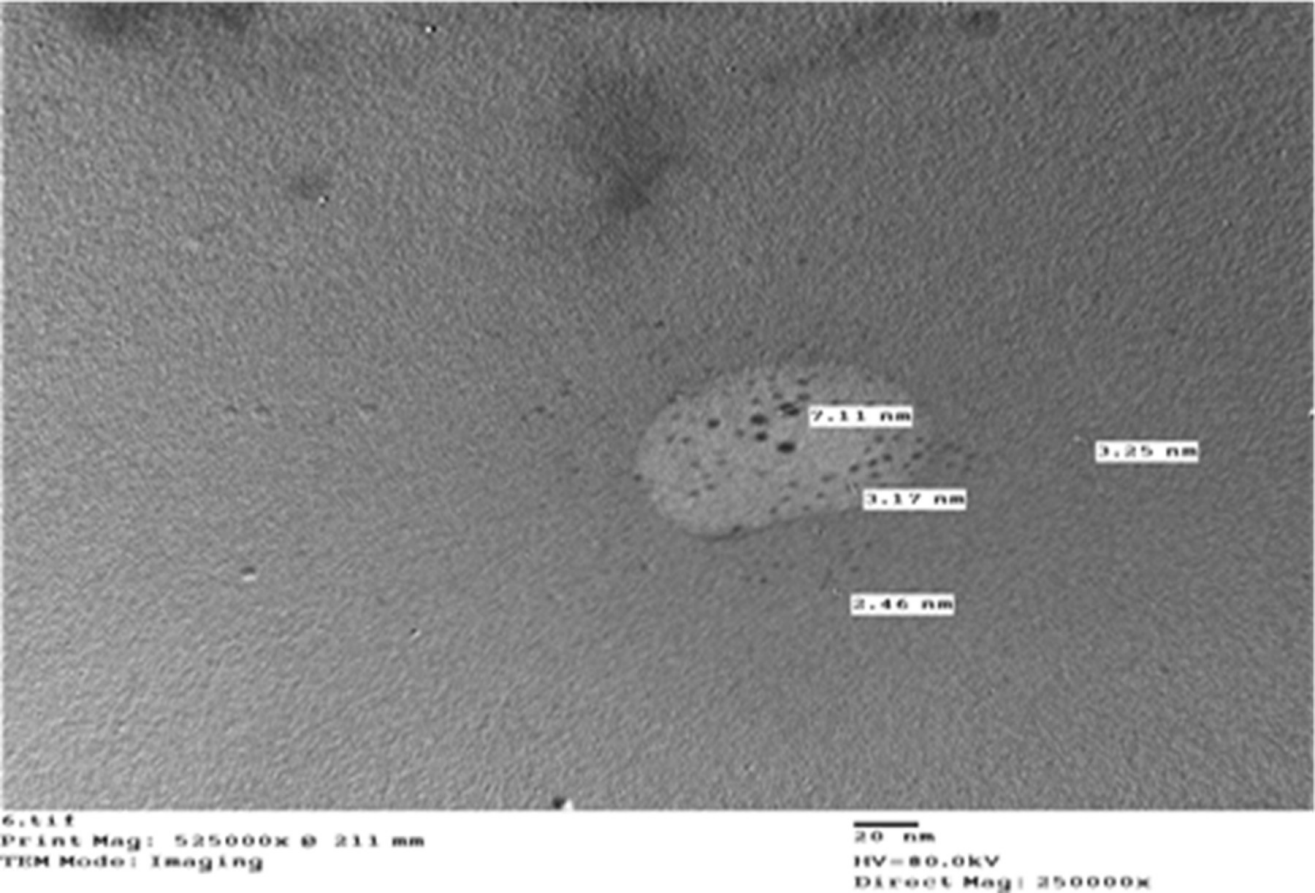
Nano-chlorine.

**Fig 2:**
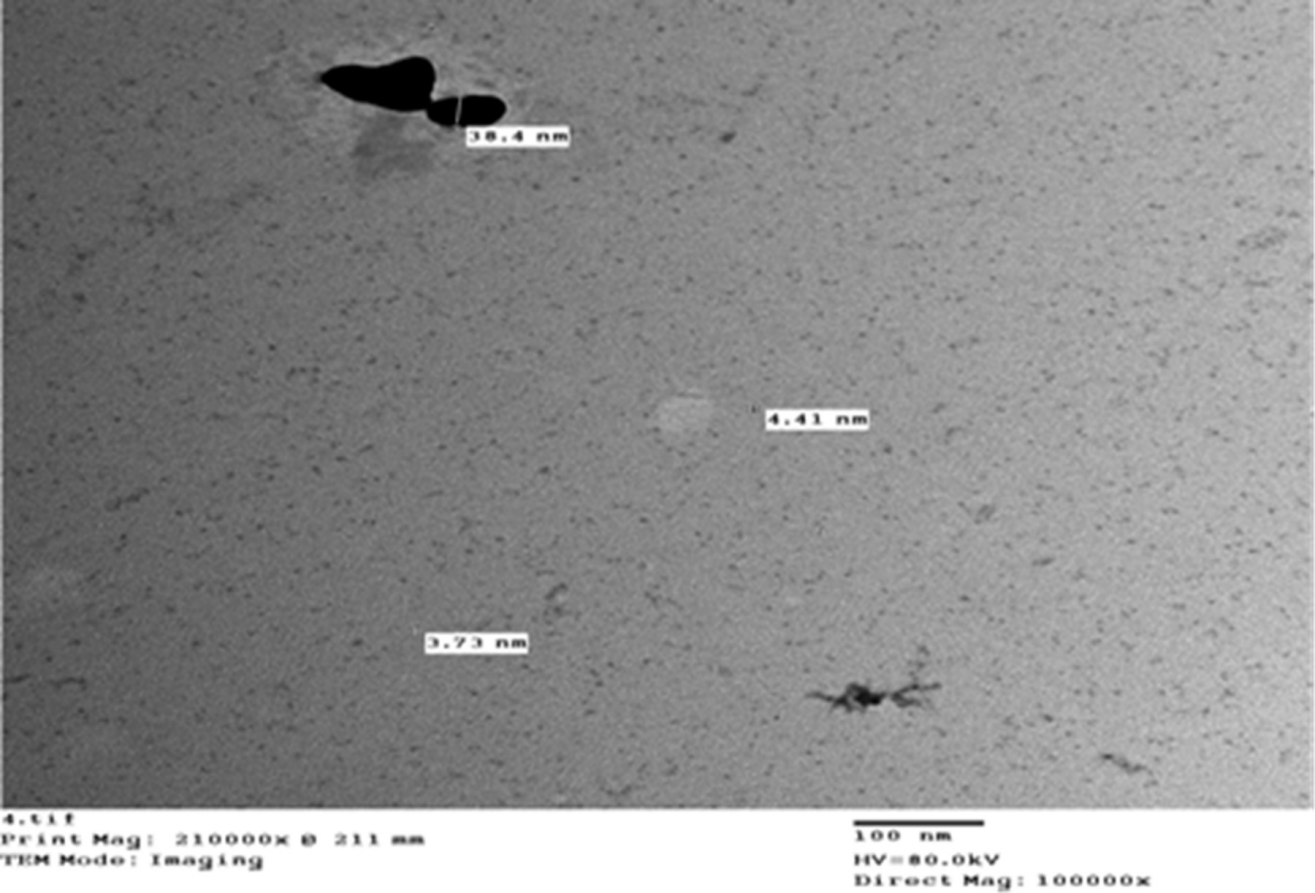
Nano-sulphur.

The nano-suspensions of chlorine (Fig 1) and sulfur (Fig 2) were achieved using top-down molecular chemical techniques. Briefly, a single drop of the nanoparticle solution was spread onto a carbon-coated copper grid, and then was posteriorly dried at room temperature for transmission electron microscope (TEM) analysis. The dimensions of the nanoparticles were established directly from the figure using Image-Pro Plus 4.5 software. The particles were irregular in shape, with dimensions of 3.99 nm for chlorine and 4.05 nm for sulfur (Table 1), as well as 98.5% purity for each element. The nanoparticles’ shape and dimension were examined using a JEOL 1010 TEM at 80 kV (JEOL, Japan).

**Table 1:** Particle size of nano-chlorine, and nano-sulphur.

Formulation	Sample number	Size (nm)	Average size (nm)
Nano-chlorine	1	3.25	3.99
	2	7.11	
	3	3.17	
	4	2.46	
Nano-sulphur	1	3.84	4.05
	2	4.41	
	3	3.73	
	4	4.22	

### Activities of chlorantraniliprole, thiocyclam, and their nano-forms

The toxicity of chlorantraniliprole, thiocyclam, and their nano-forms on the second instar larvae are presented in Table 2. The LC_50_ values were 0.058 and 9.20 mg/l for chlorantraniliprole and thiocyclam, respectively, 96 h post-treatment. In contrast, the nano-forms (nano-chlorantraniliprole and nano-thiocyclam) had significantly higher toxicities than their original compounds, with LC_50_ values of 0.015 and 4.46 mg/l, respectively (Table 2).

**Table 2:** Lethal and sublethal activity of chlorantraniliprole, thiocyclam, and their nano-form in the 2^nd^ instar larvae of *Agrotis ipsilon*.

Insecticides Treatments	^a^LC_15_ (mg/L) (95% Confidence limit)	^b^LC_50_ (mg/L) (95% Confidence limit)	^c^LC_90_ (mg/L) (95% Confidence limit)	Slope ± SE	χ^2^
Chlorantraniliprole	0.001 (0.000–0.003)	0.058 (0.010–0.159)	8.21 (2.17–141.89)	0.59 ± 0.12	4.10
Nano-Chlorantraniliprole	0.001 (0.000–0.003)	0.015 (0.006–0.038)	2.65 (0.57–48.27)	0.56 ± 0.09	3.96
Thiocyclam	0.052 (0.000–0.426)	9.209 (2.03–34.10)	1622.48 (233.63–38982.06)	0.57 ± 0.15	3.42
Nano-Thiocyclam	0.44 (0.11–0.89)	4.46 (2.87–6.78)	45.14 (23.29–154.65)	1.27 ± 0.21	4.44

^a^ LC_15_: Concentration to cause mortality in 15% of individuals.

^b^ LC_50_: Lethal concentration to cause mortality in 50% of individuals.

^c^ LC_90_: Lethal concentration to cause mortality in 90% of individuals.

### Lethal and sublethal effects on the second instar larvae

Table 3 shows the latent effect of chlorantraniliprole, thiocyclam, and their nano-forms on *A*. *ipsilon* development (from second instar larvae until the emergence) due to LC_15_ and LC_50_ exposure. The results showed elongation of the larval developmental period under all LC_50_ treatments, while pupal duration was significantly increased in larvae exposed to regular form of both insecticides. The pupation% decreased significantly after LC_15_ and LC_50_ treatment of nano-thiocyclam at 83.20 ± 4.36% and 85.88 ± 0.56%, respectively (Table 3). No differences were found in the female and male pupal weight, sex ratio, or emergence% for all treatments (Table 3).

**Table 3:** Effects of chlorantraniliprole, thiocyclam, and their nano-form in the developmental stages of *A*. *ipsilon*.

Treatments		^a^Larval duration	Pupation%	^b^Pupal duration	Pupal weight (mg)		Sex ratio%		^c^Emergence%
					Female	Male	Female	Male	
Control		20.22±0.15	93.79±3.10	17.00±0.15	0.43±0.01	0.39±0.01	43.44±3.79	56.56±3.79	98.61±1.39
Chlorantraniliprole	LC_15_	21.04^ns^±0.29	95.84^ns^±0.31	17.91**±0.16	0.40^ns^±0.01	0.39^ns^±0.01	49.55^ns^±7.58	50.45^ns^±7.58	98.61^ns^±1.39
	LC_50_	24.13****±0.46	90.16^ns^±0.15	18.48****±0.28	0.39^ns^±0.01	0.35^ns^±0.01	42.85^ns^±2.28	57.15^ns^±2.28	98.15^ns^±1.85
Nano-Chlorantraniliprole	LC_15_	22.25***±0.31	93.92^ns^±1.31	17.73^ns^±0.24	0.39^ns^±0.01	0.36^ns^±0.01	49.38^ns^±9.97	50.62^ns^±9.97	96.74^ns^±1.63
	LC_50_	22.30**** ±0.26	91.07^ns^±2.25	17.46^ns^±0.20	0.42^ns^±0.01	0.40^ns^±0.01	51.78^ns^±10.26	48.22^ns^±10.25	90.54^ns^±2.32
Thiocyclam	LC_15_	21.66*±0.34	92.60^ns^±1.23	17.77*±0.19	0.39^ns^±0.02	0.38^ns^±0.01	42.15^ns^±5.32	57.85^ns^±5.32	98.24^ns^±1.75
	LC_50_	23.30****±0.39	93.82^ns^±1.91	17.46*±0.16	0.41^ns^±0.01	0.37^ns^±0.01	46.40^ns^±1.32	53.60^ns^±1.32	98.61^ns^±1.39
Nano-Thiocyclam	LC_15_	22.52****±0.28	86.54^ns^±1.73	17.54^ns^±0.19	0.36*±0.008	0.35^ns^±0.01	42.67^ns^±4.66	57.33^ns^±4.66	96.63^ns^±1.71
	LC_50_	22.75****±0.35	82.55*±3.79	17.27^ns^±0.12	0.39^ns^±0.01	0.39^ns^±0.01	49.59^ns^±12.14	50.41^ns^±12.14	88.85*±3.38

ns is no significant different at P > 0.05

* Significant diferent at P ≤ 0.05

** significant different at P ≤ 0.01

*** significant different at P ≤ 0.001

**** significant different at P ≤ 0.0001.

^a^number of days from 2^nd^ instar larvae till pupation.

^b^number of days from the pupation till the emergence.

^c^Emergence % = Number of moths/Total number of pupae × 100.

### Fecundity and fertility

Chlorantraniliprole, thiocyclam, and their nano-forms significantly decreased the hatchability percent under LC_15_ and LC_50_ compared to the control (Table 4). After chlorantraniliprole and nano-chlorantraniliprole treatment, the hatchability percentages were 84.60, and 81.29% at LC_15_, and 73.83, and 73.08% at LC_50,_ respectively. Under thiocyclam and nano-thiocyclam treatment, the hatchability percentages were 82.64, and 78.32% at LC_15_, and 78.79, and 71.99% at LC_50_. In contrast, the number of eggs laid by one female showed no significant differences between treated and untreated larvae (Table 4).

**Table 4:** Mean fecundity and hatchability % (±SE) of *A*. *ipsilon* females after treating the 2^nd^ instar larvae with LC_15_ and LC_50_ values of chlorantraniliprole, thiocyclam, and their nano-form.

Treatments		^a^Fecundity	^b^Hatchability %
Control		539.10±13.71	90.78±0.31
Chlorantraniliprole	LC_15_	412.93^ns^±10.88	84.60^ns^±3.65
	LC_50_	282.00**±58.29	73.84**±5.53
Nano-Chlorantraniliprole	LC_15_	386.70^ns^±39.50	81.29^ns^±4.60
	LC_50_	286.3**±32.92	73.08**±0.93
Thiocyclam	LC_15_	446.60^ns^±37.99	82.64^ns^±3.31
	LC_50_	365.00^ns^±63.60	78.79^ns^±2.23
Nano-Thiocyclam	LC_15_	276.70**±15.38	78.32^ns^±2.22
	LC_50_	265.00**±77.12	71.99**±1.78

ns is no significant different at P > 0.05

* Significant diferent at P ≤ 0.05

** significant different at P ≤ 0.01

*** significant different at P ≤ 0.001

**** significant different at P ≤ 0.0001.

^a^Fecundity was estimated by counting the eggs from the first day till the sixth day (total number of eggs laid by one female).

^b^Fertility was calculated by counting of the emerged larvae from collected eggs batch.

### Activity of oxidative stress enzymes

Exposure to both insecticides and their nano-forms caused a significant increase in SOD activity after treatment with the LC_50_ of thiocyclam (40.09 U/g of protein) and nano-thiocyclam (43.0 U/g of protein) (Table 5). The SOD activity after treatment with the LC_50_ of chlorantraniliprole and that of nano-chlorantraniliprole was highly significant compared to the control treatment with 45.38 and 53.96 U/g of protein (ca. 5.44, and 6.47 fold), respectively. No significant changes relative to the control were recorded in any other treatments.

**Table 5:** Mean (±SE) of oxidative stress enzymes (SOD, CAT, glutathione reductase, and lipid peroxidase) activities of *A*. *ipsilon* after exposure of 2^nd^ instar larvae to LC_15_ and LC_50_ values of chlorantraniliprole, thiocyclam, and their nano-forms.

Treatments		Mean ± SE			
		SOD U/g of protein	CAT U/g of protein	Glutathione reductase U/g of protein	Lipid peroxidase nmol/g of protein
Control		8.33±2.4	17.95±4.74	1.42±0.10	0.23±0.07
Chlorantraniliprole	LC_15_	33.68^ns^±2.66	80.22*±4.71	11.04*±1.57	1.60^ns^±0.41
	LC_50_	45.38**±11.78	99.78**±25.06	11.76*±3.79	2.24*±0.58
Nano-Chlorantraniliprole	LC_15_	35.41^ns^±2.05	87.76*±3.95	12.56**±2.65	1.68^ns^±0.07
	LC_50_	53.96**±10.53	99.60**±12.6	14.33**±2.06	3.75****±0.57
Thiocyclam	LC_15_	24.01^ns^±1.31	76.03*±8.34	3.73^ns^±0.06	1.25^ns^±0.14
	LC_50_	40.09*±8.45	89.90**±21.81	6.41^ns^±1.63	2.02*±0.26
Nano-Thiocyclam	LC_15_	34.59^ns^±4.67	80.28*±10.40	12.67**±2.00	1.54^ns^±0.22
	LC_50_	43.0*±6.61	86.43*±12.55	14.42**±2.20	2.26*±0.68

ns is no significant different at P > 0.05

* Significant different at P ≤ 0.05

** significant different at P ≤ 0.01

*** significant different at P ≤ 0.001

**** significant different at P ≤ 0.0001.

The LC_15_ and LC_50_ of both insecticides and their nano-forms stimulated CAT activity compared to the control (Table 5). A significant increase in CAT activity was observed at the LC_15_ of chlorantraniliprole and nano-chlorantraniliprole, the LC_15_ of thiocyclam, and both the LC_15,_ and LC_50_ of nano-thiocyclam (80.22, 87.76, 76.03, 80.28 and 86.43 U/mg of protein, respectively). The highest significance was observed for the LC_50_ of chlorantraniliprole and nano-chlorantraniliprole, and the LC_50_ of thiocyclam (ca. 5.56, 5.54, and 5.0-fold, respectively).

An increase in lipid peroxidase activity was observed under exposure to the LC_50_ of nano-chlorantraniliprole with 3.75 nmol/g of protein (ca. 16.30-fold). Meanwhile, the lipid peroxidase activity after treatment with the LC_50_ of chlorantraniliprole, thiocyclam, and nano-thiocyclam was slightly significant at 2.24, 2.02, and 2.26 nmol/g of protein, respectively. No significant differences in lipid peroxidase activities were observed in any other treatments (Table 5).

Exposure to all investigated insecticides and their nano-forms resulted in significant stimulation of GR activity in the LC_15_ and LC_50_ of chlorantraniliprole (11.4 and 11.76 U/g of protein) (Table 5). GR activity was significantly high under the LC_15_ and LC_50_ nano-chlorantraniliprole treatments at 8.84 and 10.09-fold, respectively and under the LC_15_ and LC_50_ nano-thiocyclam treatments at 8.92, and 10.15-fold, respectively. No significant changes in enzyme activity was observed for the LC_15_ and LC_50_ thiocyclam treatments (3.73 and 6.41 U/g of protein) compared to the control.

### Correlation between development, reproductive, and enzyme activity

**Fig 3:**
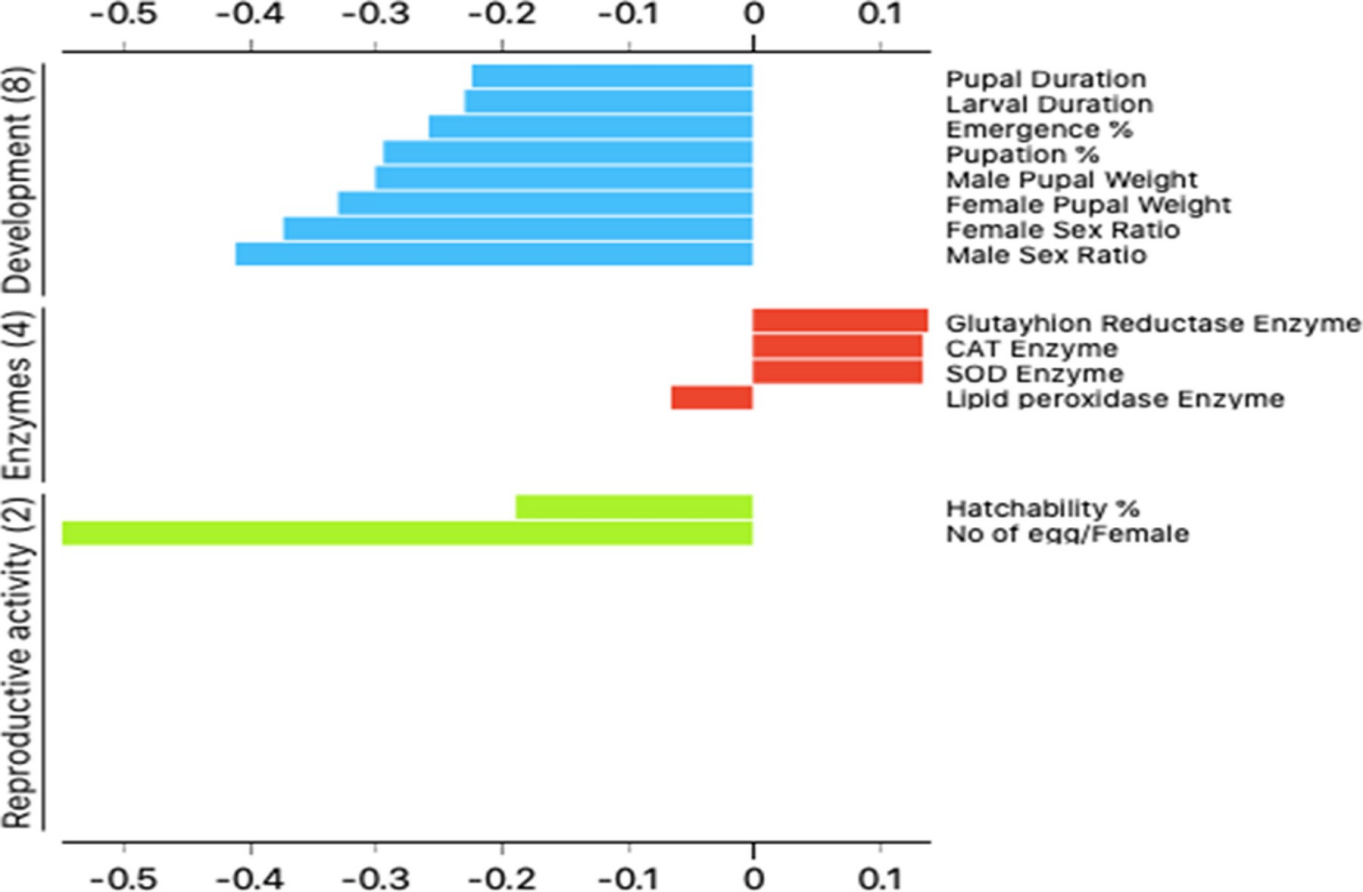
Plot of Silhouette analysis values for clustering of reproductive activity, enzymes and developmental variables. On the y-axis each cluster are ordered by decreasing silhouette value. The silhouette value can range between −1 and 1.

**Fig 4:**
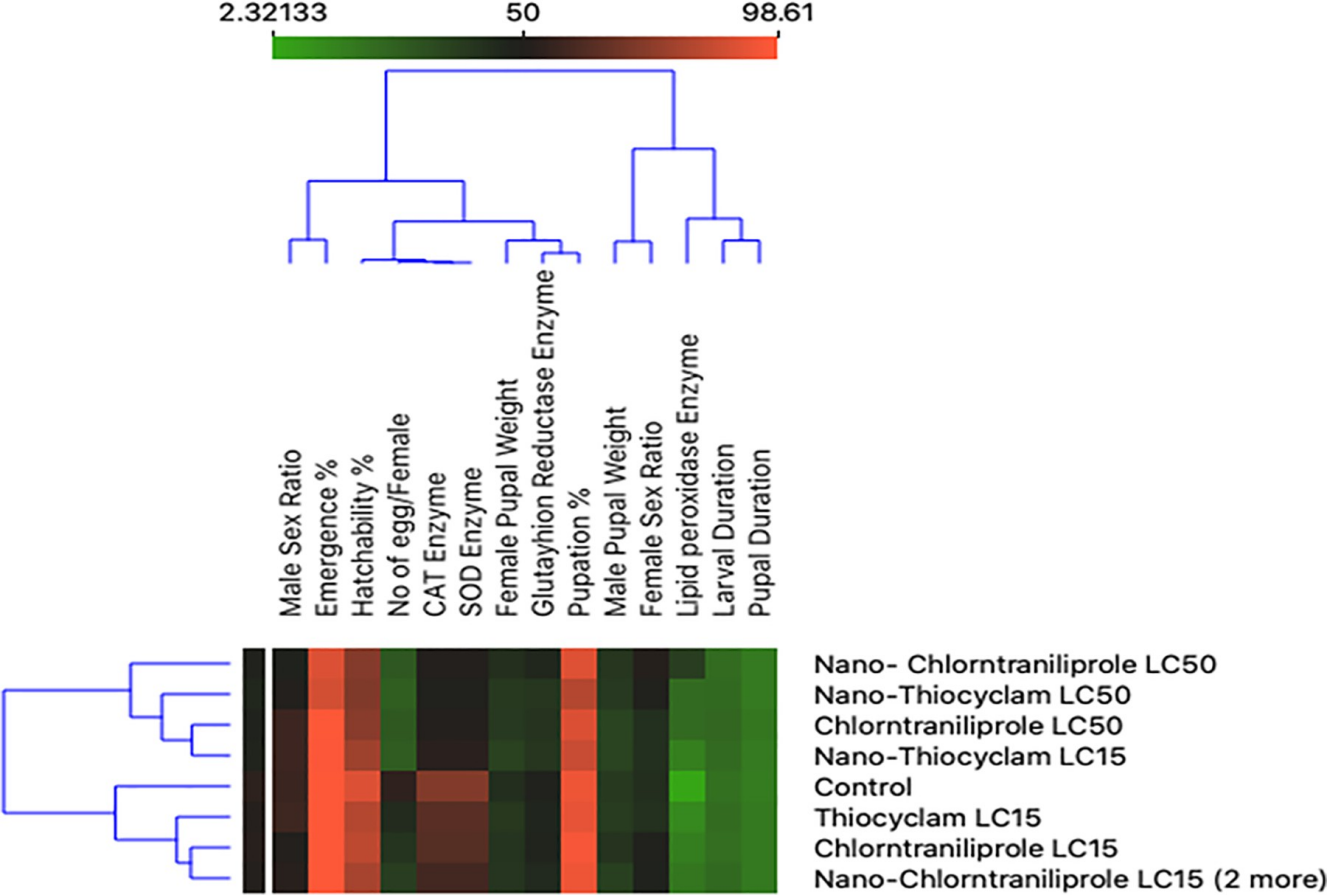
Two-dimensional heatmap visualization shows the interaction between the treatments and (A) the eight developmental parameters (B) the two reproductive activity parameters (C) the four enzymes parameters.

**Fig 5:**
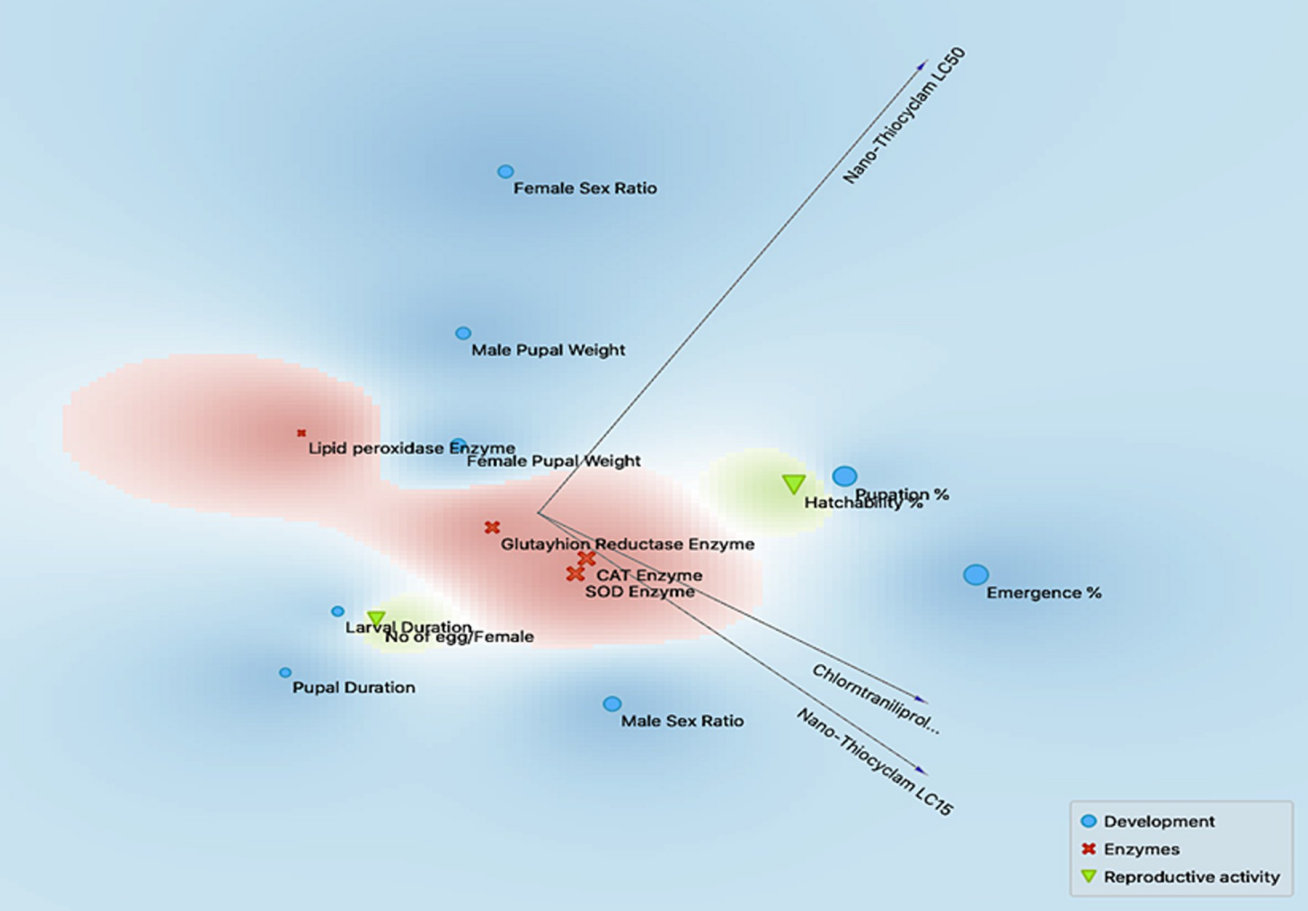
Multidimensional preference analysis plot summarizing the interrelationships amongst treatments, parameters, and classes.

The plots for silhouette analysis were calculated based on the Euclidean distance metric to assess the cluster quality of the treatments based on reproductive activity, enzymes, and developmental measurements via cluster distance tests within and between each cluster (Fig 3). The results revealed that all parameters, except GR, SOD, and CAT enzymes, exhibited negative values, thus indicating that the clusters were mostly similar, and that the cluster configuration may have too few clusters. Meanwhile, two-dimensional heatmap plotting based on all parameters clustered the LC_15_ of thiocyclam, chlorantraniliprole, and nano-chlorantraniliprole as the most similar to the control (1^st^ cluster), whereas the LC_50_ of nano-chlorantraniliprole, nano-thiocyclam, chlorantraniliprole, and the LC_15_ of nano-thiocyclam were grouped in the second cluster as less-similar relative to the control (Fig 4). Moreover, multidimensional preference analysis was performed to summarize the interrelationships of all treatments, parameters, and classes (Fig 5). The plot shows that the LC_50_ of nano-thiocyclam, chlorantraniliprole, and the LC_15_ of nano-thiocyclam deviated the most compared to the control. GR, SOD, and CAT enzymes revealed almost the same pattern.

### ISSR analysis

**Fig 6:**
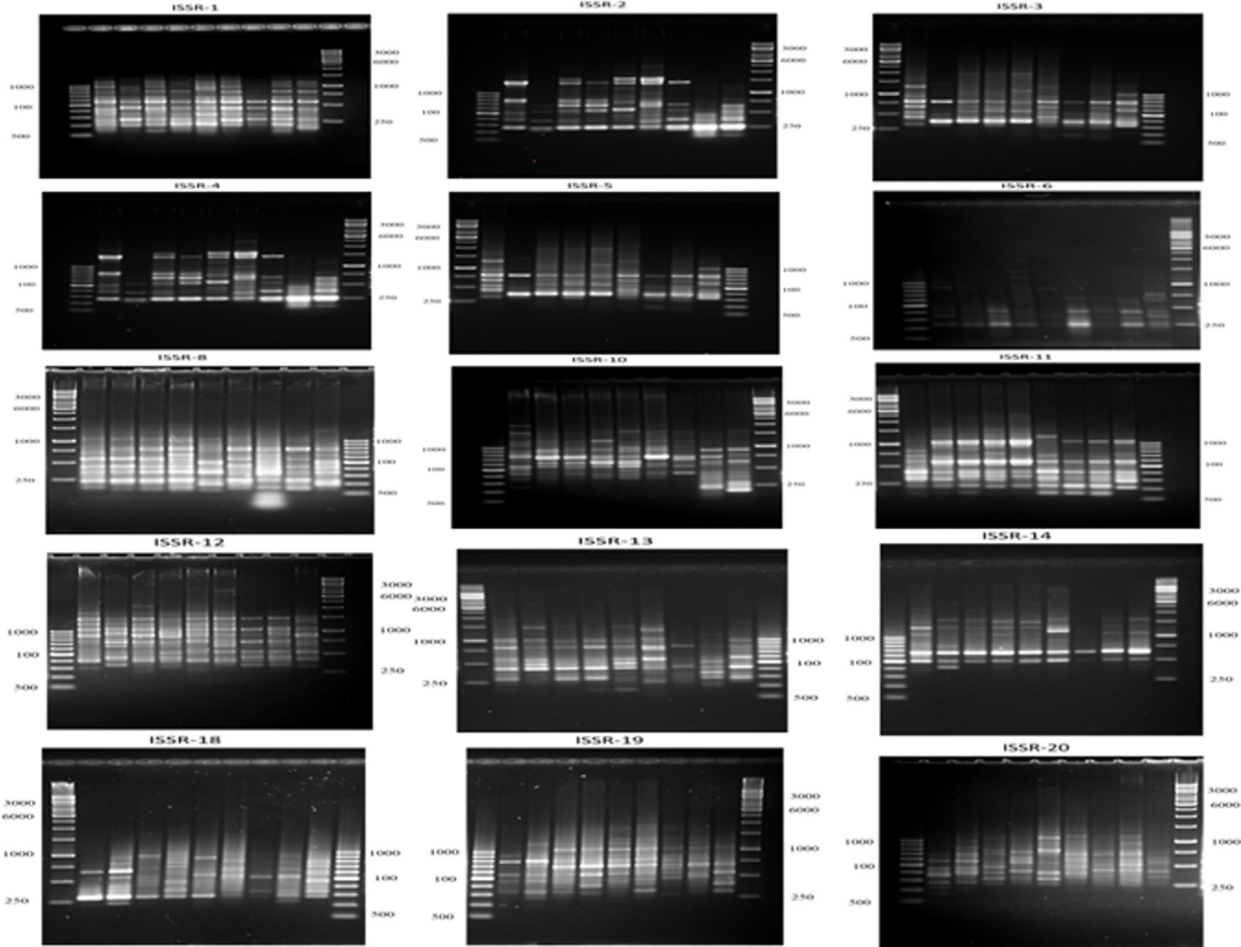
A representative agarose gel where PCR products of the 15 ISSR primers for the nine treatments.

To determine the mutagenic levels of the chlorantraniliprole, thiocyclam, and their nano-forms on the DNA of the second instar larvae, the ISSR marker system was used. The DNA of the untreated second instar larvae and all other treatments were amplified using 15ISSR primers (Fig 6). The 15 ISSR primers yielded a total of 252 scorable amplicons with an average of 16.8 bands/primer (Table 6). The number of amplified DNA fragments per primer ranged from 11 bands (primer ISSR-18) to 21 bands (primers ISSR-10 and ISSR-12). The number of polymorphic bands per primer ranged from 7 bands (primer ISSR-1) to 19 bands (primer ISSR-5). A narrow range of the expected heterozygosity values was observed between 0.37 to 0.50. Notably, 13 out of the 15 ISSR primers showed values near 0.50. The polymorphism information content almost revealed the same pattern for all primers, with values ranging from 0.30 to 0.37. The effective multiplex ratio values were almost all high, with values ranging from 6.00 to 12.67. In contrast, the marker index values were shallow (near to 0.01). The discriminating power values ranged from 0.44 to 0.86. The resolving power values ranged between 4 to 10. The primers ISSR-2 and ISSR-5 showed the highest-resolving power value (10.44) among all the ISSR primers (Table 6). The highest GS was observed between the LC_50_ of chlorantraniliprole and the LC_15_ of thiocyclam. The lowest GS was determined to be between the LC_50_ of nano-chlorantraniliprole, and nano-thiocyclam (Table 7).

**Table 6:** Primer names, number of total bands, polymorphic bands, percentage of polymorphism and markers efficiency parameters of ISSR primers.

Primer Name	No. of Polymorphic Bands	No. of Monomorphic Bands	Total No. of bands	% of polymorphism	H	PIC	E	H.av	MI	D	R
ISSR-1	7	6	13	53.8	0.37	0.30	9.78	0.00	0.03	0.44	4.00
ISSR-2	15	2	17	88.2	0.50	0.37	8.56	0.00	0.03	0.75	10.44
ISSR-3	18	1	19	94.7	0.50	0.37	9.11	0.00	0.03	0.77	10.22
ISSR-4	15	1	16	93.8	0.50	0.37	7.44	0.00	0.03	0.79	9.56
ISSR-5	19	1	20	95.0	0.50	0.37	9.89	0.00	0.03	0.76	10.44
ISSR-6	16	2	18	88.9	0.47	0.36	6.67	0.00	0.02	0.86	7.56
ISSR-8	13	2	15	86.7	0.41	0.33	10.67	0.00	0.03	0.50	5.56
ISSR-10	18	3	21	85.7	0.49	0.37	11.89	0.00	0.03	0.68	8.22
ISSR-11	16	4	20	80.0	0.46	0.36	12.67	0.00	0.03	0.60	7.11
ISSR-12	18	3	21	85.7	0.49	0.37	12.00	0.00	0.03	0.67	8.00
ISSR-13	13	2	15	86.7	0.49	0.37	8.67	0.00	0.03	0.67	5.33
ISSR-14	10	2	12	83.3	0.49	0.37	6.67	0.00	0.03	0.69	4.67
ISSR-18	10	1	11	90.9	0.50	0.37	6.00	0.01	0.03	0.71	7.33
ISSR-19	15	2	17	88.2	0.47	0.36	10.44	0.00	0.03	0.62	7.78
ISSR-20	17	0	17	100.0	0.50	0.37	7.78	0.00	0.03	0.79	9.33
Total	220	32	252	252							
Average	14.66	2.13	16.8								

**Table 7:** Genetic similarities between the nine treatments based on Jaccard’s similarity coefficient based on ISSR primers data.

	Control	C15	C50	T15	T50	Cn15	Cn50	Tn15	Tn50
**Control**	100%	--	--	--	--	--	--	--	--
**C15**	49%	100%	--	--	--	--	--	--	--
**C50**	56%	50%	100%	--	--	--	--	--	--
**T15**	54%	51%	66%	100%	--	--	--	--	--
**T50**	54%	46%	65%	62%	100%	--	--	--	--
**Cn15**	58%	51%	65%	56%	60%	100%	--	--	--
**Cn50**	41%	47%	41%	44%	42%	52%	100%	--	--
**Tn15**	50%	54%	51%	50%	47%	55%	48%	100%	--
**Tn50**	46%	43%	52%	58%	44%	47%	37%	50%	100%

Symbols: C; Control, C15; chlorntraniliprole LC_15_, C50; chlorntraniliprole LC_50_, Cn15; nano-chlorntraniliprole LC_15_, Cn50; Nano-chlorntraniliprole LC_15_, T15; thiocyclam LC_15_, T50; thiocyclam LC_50_, Tn15; nano-thiocyclam LC_15_ and Tn50; nano-thiocyclam LC_50_.

### Analysis of molecular phylogeny

**Fig 7:**
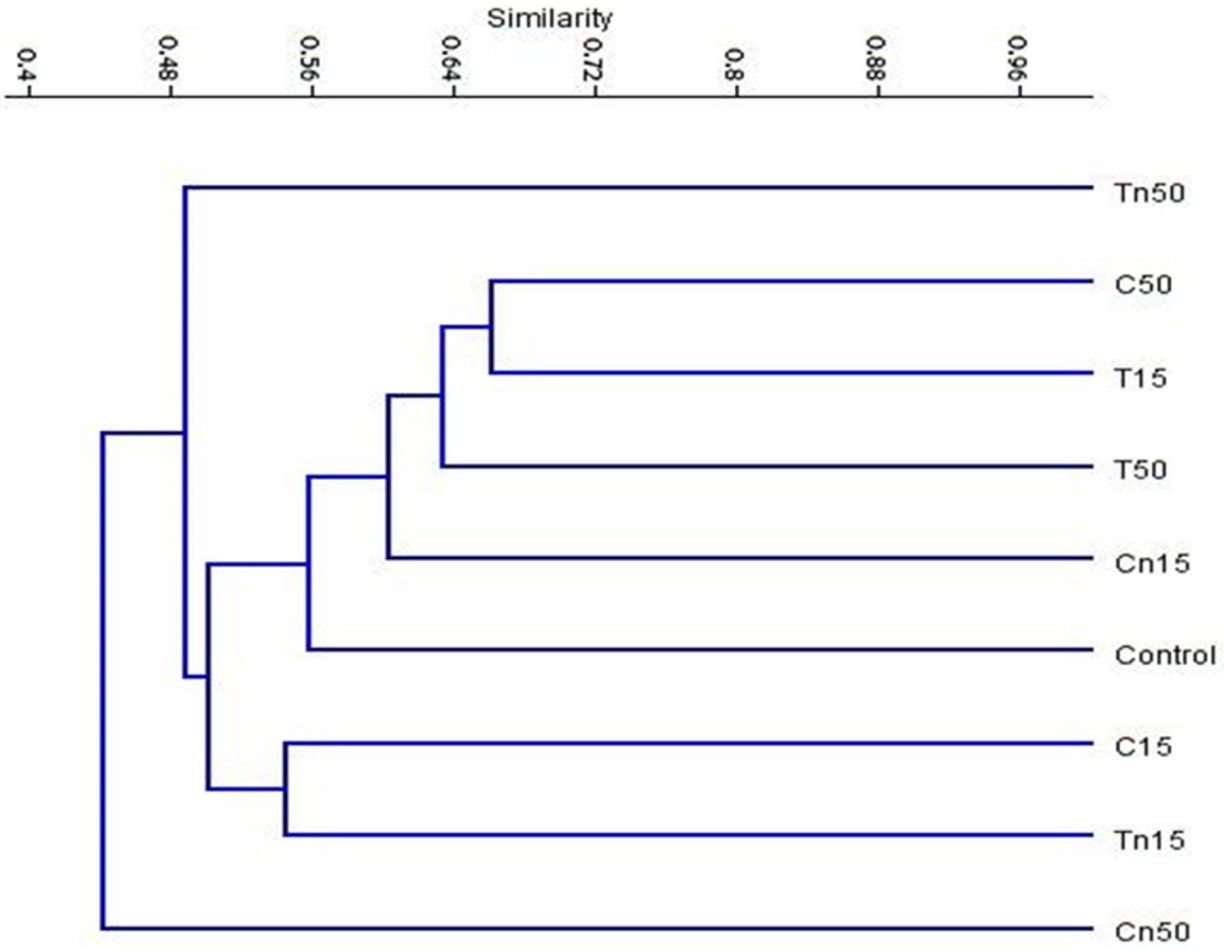
UPGMA cluster analysis based on Jaccard’s similarity coefficient of ISSR analysis of the nine treatments: C; Control, C15; chlorntraniliprole LC_15_, C50; chlorntraniliprole LC_50_, Cn15; nano-chlorntraniliprole LC_15_, Cn50; nano-chlorntraniliprole LC_50_, T15; thiocyclam LC_15_, T50; thiocyclam LC_50_, Tn15; nano-thiocyclam LC_15_ and Tn50; nano-thiocyclam LC_50_.

**Fig 8:**
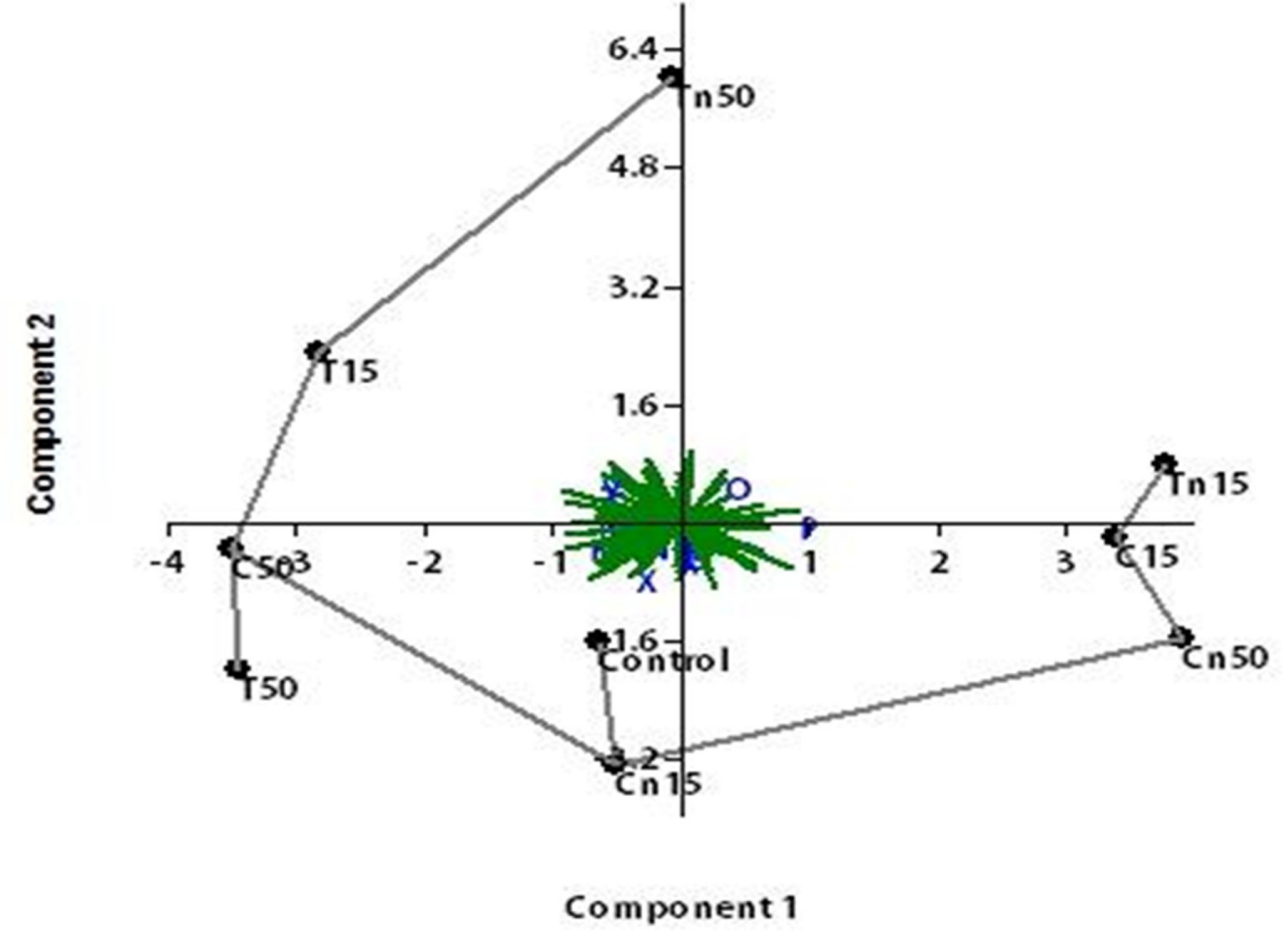
A representative agarose gel where PCR products of the 15 ISSR primers for the nine treatments.

A dendrogram based on the UPGMA cluster analyses of the ISSR data was constructed for the nine treatments (Fig 7). The dendrogram was comprised of two main clusters: the first cluster included only the LC_50_ of chlorantraniliprole, while the second cluster comprised two sub-clusters. The first sub-cluster consisted of only the LC_50_ of nano-thiocyclam, and the second sub-cluster involved two major groups. The first major group included the LC_15_ treatments of chlorantraniliprole, and nano-thiocyclam. The second major group consisted of the control, the LC_15_ treatments of chlorantraniliprole, nano-chlorantraniliprole, and thiocyclam_,_ and the LC_50_ of thiocyclam. Furthermore, the PCA analysis of the ISSR data revealed highly similar results to the cluster analysis. The PCA results indicated that the LC_15_ of nano-chlorantraniliprole and the LC_50_ of thiocyclam were most similar to the control (Fig 8).

## Discussion

Insecticide efficacy depends on the mode of action, insect species, developmental stage, application methods, and the number of days post-treatment [50]. Thus, pesticide nano-formulations may change the toxicity or residue activity of insecticides, and reducing its application frequency, according to the Environmental Protection Agency’s recommendation [51]. In the present study, we investigated the effects of the lethal and sublethal concentrations of thiocyclam, chlorantraniliprole, and their nano-forms on *A*. *ipsilon* larval and pupal duration, larval mortality, adult emergence, reproductive activity, sex ratio, and oxidative stress enzymes with particular emphasis on DNA mutagenicity.

Chlorantraniliprole has a novel mode of action [52] that activates insect ryanodine receptors (RyRs), leading to paralysis and mortality in sensitive species [53]. Thiocyclam is a broad-spectrum nereistoxin antagonist that blocks the transmission of cholinergics [52], resulting in insect death. Our results revealed that *A*. *ipsilon* larvae were more susceptible to the nano-forms than the regular forms for both insecticides (Table 2). This might be due to (1) the nano chlorine within chlorantraniliprole, since chlorine is a non-selective oxidant with a number of effects on the living biota (e.g., reacts with a variety of cellular components, deactivates enzymatic active sites, decreases the biological functions of proteins, and produces deleterious effects on DNA [54]. In some cases, different layers of protein present in insects, larvae or even spores provide protection against chemical attacks, including chlorine, but we speculate that nano-chlorine has a greater effect on the cytoplasmic membrane permeability, causing the loss of refractivity, separating the spore coats from the cortex, extensively discharging Ca+, dipicolinic acid, and DNA, and finally causing lysis to occur, which can lead to cell death and growth inhibition [55]. The increased susceptibility to the nano-forms may also be due to (2) the nano-sulfur within thiocyclam, since sulfur destroys an insect’s normal energy-producing bodily functions [56]. Normal pesticides containing sulfur have some caveats, because sulfur can damage plants during hot, dry weather and is incompatible with some other pesticides. In addition, sulfur should not be used on plants that have been sprayed with horticultural oils, as the induced reaction can damage foliage. Sulfur is non-toxic to humans and animals, unless ingested [57].

Similarly, thiocyclam is a highly effective insecticide against the field strain [58,59] of *Tuta absoluta* (Meyrick) (9.98 mg/L). In contrast, *A*. *ipsilon* larvae were more susceptible to chlorantraniliprole and its nano-form than thiocyclam (Table 1). Likewise other lepidopteran pests, including *Helicoverpa armigera* (Hübner), *Spodoptera exigua* (Hübner), and *Spodoptera littoralis* (Boisd.) [31,60,61] are also susceptible to this insecticide. The chlorantraniliprole showed harmful activity toward the third instar larvae of *A*. *ipsilon* [62], whereas its LC_50_ was 0.187 μg/g 72 h post-treatment. This variation in susceptibility to chemical insecticides could be due to nutrition type, size, and physiological status of the host [63].

Sublethal effects of insecticides could be considered a common toxicological phenomenon and represent a secondary effect of insecticide application [62,64]. This phenomenon may occur in insects due to exposure to degraded insecticide after its initial application on crops [31]. In our results, the developmental rates of the larvae and pupae were substantially extended (Table 2) after treating the 2^nd^ instar larvae of *A*. *ipsilon* with the original form of both insecticides compared to their nano-forms. These results agree with He et al. [62], Lutz et al. [65], El-Dewy [66], and Han et al. [67], whom found that the development of *A*. *ipsilon*, *Plutella xylostella* (Linnaeus), *S*. *littoralis*, and *Spodoptera cosmioides* (Walker) larvae was prolonged by low concentrations of chlorantraniliprole. In this study, both insecticides and their nano-forms did not show any significant differences in other biological parameters except for the LC_50_ of nano-thiocyclam, which exhibits a significant difference in pupation% and emergence%. Additionally, the nano-form of both insecticides at LC_50_ showed a significant difference in the number of eggs laid per female and fertility.

In the last decade, pesticide-induced oxidative stress has been a focus of toxicological research as a possible toxicity mechanism that causes a final manifestation of a pro-oxidant and antioxidant defense mechanism imbalance [68]. Pesticide intoxication causes a derangement of different antioxidant mechanisms in various tissues [68]; however, exposure to sublethal insecticide concentrations can induce oxidative stress enzymes to increase (e.g., SOD, CAT, and GR) [69]. Our results indicate that the CAT activity was significantly increased in both the insecticides and their nano-forms. Significantly increased SOD activity was observed in the LC_50_ of both insecticides and their nano-forms. Also, significantly higher GR activities were observed in nano-chlorantraniliprole and nano-thiocyclam. The antioxidant enzyme systems, such as CAT or SOD, play an essential role in detoxifying harmful agents to counter the damaging effects of ROS [70].

Similar to the elevated antioxidant levels, lipid peroxide activity was significantly increased in MDA, which is the main oxidation result from the peroxidation of unsaturated fatty acids. This represents the oxidative effect on different organisms [71,72] in the LC_50_ of both insecticides and their nano-forms. We observed no significant differences between the insecticides and their nano-forms at the lowest tested concentration (LC_15_), which reflects the protective effects of antioxidants in *A*. *ipsilon* larvae. The antioxidant system can reduce MDA levels, which contributes to the accumulation of active oxygen and inhibits antioxidase activity [73]. The accumulation of MDA can be reduced by antioxidant enzymes, such as SOD and CAT. Other studies have demonstrated that toxic oxygen metabolism can mediate lipid peroxidation [74].

The ISSR-PCR technique was used as an effective marker to investigate the genetic mutagenicity levels between the second instar larvae subjected to different insecticidal treatments (chlorantraniliprole, thiocyclam and their nano-forms) compared to the untreated control. One of the benefits of ISSR is that it performs a qualitative evaluation of DNA variability through the differences in amplification profiles [44]. Many studies reported that the exposure of insects to particular insecticides at different concentration levels might lead to damages or changes in genomic DNA sequences (e.g., insertions, deletions, substitutions, or rearrangements), resulting in changes in the ISSR profile (e.g., the presence or absence of certain bands or variations of the band intensity) [25]. In addition, the possible occurrence of point mutations at the oligonucleotide annealing site may cause the absence of bands due to the loss of a priming site [75]. In this study, 15 ISSR primers were used to detect genetic mutagenicity levels and screen the degree of polymorphism between treated and untreated larvae. Furthermore, to establish the relationship between the insecticides, the ISSR study results were used to create a dendrogram. The results showed that the lowest mutagenic insecticidal effects on the insect DNA was observed in the LC_15_ of nano-chlorantraniliprole treatment compared to the control. In contrast, the highest mutagenic effect was observed in the LC_50_ of nano-chlorantraniliprole, followed by the LC_50_ of nano-thiocyclam compared to the control. The PCA analysis of ISSR data revealed a consistent result to that obtained by the dendrogram topology. Moreover, it was found that the LC_15_ of nano-chlorantraniliprole and the LC_50_ of thiocyclam exhibited almost the same mutagenic effects as the control.

It is expected that the polymorphic differences observed in the ISSR patterns may be attributed to changes in primer binding sites or DNA structures, or to DNA damage caused by insecticide exposure. In addition, it could also be due to the blocking of DNA replication, the presence of a large number of chromosomal lesions, such as large rearrangements (e.g., deletion, inversion, or translocation), or un-repaired DNA damage due to direct exposure to different insecticidal treatments [76]. The ISSR studies demonstrated their ability and effectiveness to detect DNA damage and changes caused by the studied insecticides.

## Conclusion

In summary, we developed insecticide nanometerization to improve the biological activity of conventional insecticides. Our obtained results represent a promising step toward developing safe and efficient nano-insecticides. Further investigations are still needed to determine their effects on the environment. To the best of our knowledge, this research is a pioneer case-study that examined and analyzed the genome-wide DNA mutability, biochemical effects, and toxicity levels of chlorantraniliprole, thiocyclam, and their nano-forms for the control of *A*. *ipsilon*.

## References

[1] Binning RR, Coats J, Kong X, Hellmich RL. Susceptibility to *Bt* proteins is not required for *Agrotis ipsilon* aversion to *Bt* maize. Pest Manag Sci. 2015; 71: 601–606. doi: 10.1002/ps.3901 25186105 PMC4407924

[2] Abd El-Aziz SE, Omer EA, Sabra AS. Chemical composition of *Ocimum americanum* essential oil and its biological effects against, *Agrotis ipsilon*, (Lepidoptera: Noctuidae). Res J Agric Boil Sci. 2007; 3: 740–747.

[3] Amin AH, Bayoumi AE, Dimetry AZ, Youssef Efficiency of Nano-formulations of neem and peppermint oils on the bionomics and enzymatic activities of *Agrotis ipsilon* larvae (Lepidoptera: Noctuidae). J Nat Resou. 2019; 4: 102.

[4] Guedes C, Siqueira A. The tomato borer *Tuta absoluta*: insecticide resistance and control failure. Plant Sci Rev. 2012; 7: 1–7.

[5] Kah M. Nanopesticides and nanofertilizers: emerging contaminants or opportunities for risk mitigation?. Front Chem. 2015; 3: 64. doi: 10.3389/fchem.2015.00064 26636068 PMC4644784

[6] Worrall EA, Hamid A, Mody KT, Mitter N, Pappu HR. Nanotechnology for plant disease management. J Agron. 2018; 8: 285.

[7] Yan S, Cheng WY, Han ZH, Wang D, Yin MZ, Du XG, et al. Nanometerization of thiamethoxam by a cationic star polymer nanocarrier efficiently enhances the contact and plant-uptake dependent stomach toxicity against green peach aphids. Pest Manag Sci. 2021; 77: 1954–1962. doi: 10.1002/ps.6223 33314574

[8] Kah M, Hofmann T. Nanopesticide research: current trends and future priorities. Environ Inter. 2014; 63: 224–235. doi: 10.1016/j.envint.2013.11.015 24333990

[9] Pérez-de-Luque A, Rubiales D. Nanotechnology for parasitic plant control. Pest Manag Sci. 2009; 65: 540–545. doi: 10.1002/ps.1732 19255973

[10] Chaturvedi M, Molino Y, Sreedhar B, Khrestchatisky M, Kaczmarek L. Tissue inhibitor of matrix metalloproteinases-1 loaded poly (lactic-co-glycolic acid) nanoparticles for delivery across the blood-brain barrier. Int J Nanomedicine. 2014; 9: 575–588. doi: 10.2147/IJN.S54750 24531257 PMC3901738

[11] Kandil MA, Abdel-kerim RN, Moustafa MAM. Lethal and sublethal effects of bio-and chemical insecticides on the tomato leaf miner, *Tuta absoluta* (Meyrick) (Lepidoptera: Gelechiidae). Egypt J Biol Pest Control. 2020; 30: 1–7.

[12] Kodrík D, Bednářová A, Zemanová M, Krishnan N. Hormonal regulation of response to oxidative stress in insects. Inter J of Mol Sci. 2015; 16: 25788–25816. doi: 10.3390/ijms161025788 26516847 PMC4632827

[13] Martindale JL, Holbrook NJ. Cellular response to oxidative stress: signaling for suicide and survival. J Cell Physiol. 2002; 192: 1–15. doi: 10.1002/jcp.10119 12115731

[14] Felton GW, Summers CB. Antioxidant systems in insects. Arch Insect Biochem Physiol. 1995; 29: 187–197. doi: 10.1002/arch.940290208 7606043

[15] Rudnev II. Antioxidant system of black sea animals in early development. Comp Biochem Physiol. 1999; 122: 265–271. 10.1016/s0742-8413(98)10121-4 10190054

[16] Weirich GF, Collins AM, Williams VP. Antioxidant enzymes in the honey bee, *Apis mellifera*. Apidologie. 2002; 33: 3–14.

[17] Afiyanti M, Che H-J. Gatalase activity is modulated by calcium and calmodulin in detached mature leaves of sweet potato. J Plant Physiol. 2014; 171: 35–47. 10.1016/j.jplph.2013.10.003 24331417

[18] Yoshida K, Kaothien P, Matsui T, Kawaoka A, Shinmyo A. Molecular biology and application of plant peroxidase genes. Appl Microbiol Biotechnol. 2003; 60: 665–670. doi: 10.1007/s00253-002-1157-7 12664144

[19] Couto N, wood J, Barder J. The role of glutathione reductase and related enzymes on cellular redox homoeostasis network. Free Radic Biol Med. 2016; 95: 27–42. doi: 10.1016/j.freeradbiomed.2016.02.028 26923386

[20] Board PG, Menon D. Glutathione transferases, regulators of cellular metabolism and physiology. Biochim Biophys Acta Gen Subj. 2013; 1830: 3267–3288. doi: 10.1016/j.bbagen.2012.11.019 23201197

[21] Parsons BJ, Newbury HJ, Jackson MT, Ford-Lloyd BV. Contrasting genetic diversity relationships are revealed in rice (*Oryza sativa* L.) using different marker types. Mol Breed 1997; 3: 115–125.

[22] Van Droogenbroeck B, Kyndt T, Maertens I, Romeijn-Peeters E, Scheldeman X, Romero-Motochi JP, et al. Phylogeneticanalysis of the highland papayas (Vasconcellea) and *alliedgenera* (Caricaceae) using PCR-RFLP. Theor Appl Genet. 2004; 108: 1473–1486. doi: 10.1007/s00122-003-1575-7 14752605

[23] Joshi P, Dhawan V. Assessment of genetic fidelity of micropropagated *Swertia chirayita* plantlets by ISSR marker assay. Biol. Plant.2007, 5, 22–26.

[24] Perez de Castro A, Blanca JM, Diez MJ, Vinals FN. Identification of a CAPS marker tightly linked to the tomato leaf curl disease resistance gene Ty-1 in tomato. Eur J Plant Pathol. 2007; 117: 347–356.

[25] Lindroth EJ. Population genetics of the western bean cutworm (*Striacosta albicosta* Smith) across the United States. Ann Entomol Soc Am. 2011; 105: 685–692.

[26] Sharma K, Agrawal V, Gupta S, Kumar R, Prasad M. ISSR marker-assisted selection of male and female plants in a promising dioecious crop: jojoba (*Simmondsia chinensis*). Plant Biotechnol Rep. 2008; 2: 239–243.

[27] Hundsdoerfer AK, Wink M. New source of genetic polymorphisms in Lepidoptera. Z Naturforsch. 2005; 60 c: 618–624. doi: 10.1515/znc-2005-7-818 16163839

[28] De Oliveira JL, Campos EnVR, Gonçalves da Silva CM, Pasquoto T, Lima R, Fraceto LF. Solid lipid nanoparticles co-loaded with simazine and atrazine: Preparation, characterization, and evaluation of herbicidal activity. J Agric Food Chem. 2015; 63: 422–432. doi: 10.1021/jf5059045 25537071

[29] Cota-Arriola O, Onofre Cortez-Rocha M, Burgos-Hernández A, Marina Ezquerra-Brauer J, Plascencia-Jatomea M. Controlled release matrices and micro/nanoparticles of chitosan with antimicrobial potential: Development of new strategies for microbial control in agriculture. J Sci Food Agric. 2013; 93; 1525–1536. doi: 10.1002/jsfa.6060 23512598

[30] Xu ZP, Stevenson GS, Lu C-Q, Lu G-Q, Bartlett PF, Gray PP. Stable suspension of layered double hydroxide nanoparticles in aqueous solution. J Am Chem. Soc. 2006; 128: 36–37. doi: 10.1021/ja056652a 16390109

[31] Moustafa MAM, Fouad EA, Abdel‑Mobdy Y, Hamow KÁ, Mikó Z, Molnár BP, et al. Toxicity and sublethal effects of chlorantraniliprole and indoxacarb on *Spodopteralittoralis*(Lepidoptera: Noctuidae). Appl Entomol Zool. 2021; 56: 115–124.

[32] Hamada HM, Awad M, EL-Hefny M, Moustafa MMA. Insecticidal activity of garlic (*Allium sativum*) and ginger (*Zingiber officinale*) oils on the cotton leafworm, *Spodoptera littoralis* (Boisd) (Lepidoptera: Noctuidae). Afr Entomol 2018; 26: 84–94.

[33] Moustafa MMA, Kákai Á, Awad M, Fónagy A. Sublethal effects of spinosad and emamectin benzoate on larval development and reproductive activities of the cabbage moth, *Mamestra brassica*e L. (Lepidoptera: Noctuidae). Crop Prot. 2016; 90: 197–204.

[34] Misra HP, Fridovich I. The role of superoxide anion in the autoxidation of epinephrine and a simple assay for superoxide dismutase. J Biol Chem. 1972; 247: 3170–3175. 4623845

[35] Aebi H. Catalase in vitro. Meth Enzymol 1984; 105: 121–126. doi: 10.1016/s0076-6879(84)05016-3 6727660

[36] Ohkawa H, Ohishi N, Yagi K. Assay for lipid peroxides in animal tissues by thiobarbituric acid reaction. Anal Biochem.1979; 95: 351–358. doi: 10.1016/0003-2697(79)90738-3 36810

[37] Goldberg DM, Spooner RJ. Glutathione reductase. Meth Enzymol. 1983; 3: 258–265.

[38] Atia MA, Sakr MM, Mokhtar MM, Adawy SS. Development of sex-specific PCR-based markers in date palm. Methods Mol Biol. 2017a; 1638: 227–244. 28755227 10.1007/978-1-4939-7159-6_19

[39] Atia MA, Sakr MM, Adawy SS. Assessing date palm genetic diversity using different molecular markers. Methods Mol Biol. 2017b; 1638: 125–142. 28755220 10.1007/978-1-4939-7159-6_12

[40] Rousseeuw PJ. Silhouettes: a graphical aid to the interpretation and validation of cluster analysis. J. Comput. Appl. Math. 1987; 20: 53–65.

[41] Wickelmaier F. An introduction to MDS. SQRU. 2003; 46: 1–26.

[42] Abouseadaa HH, Atia MA, Younis IY, Issa MY, Ashour HA, Saleh I, et al. Gene-targeted molecular phylogeny, phytochemical profiling, and antioxidant activity of nine species belonging to the family Cactaceae. Saudi Boil Sci. 2020; 27: 1649–58. 10.1016/j.sjbs.2020.03.007 PMC7253903 32489307

[43] Jaccard P. Nouvelles recherché sur la distribution florale. Bull. Soc. Vaud. Sci. Nat. 1908, 44, 223–70.

[44] Atia MA, Osman GH, Elmenofy WH. Genome-wide in silico analysis, characterization and identification of microsatellites in *Spodoptera littoralis* multiple nucleopolyhedrovirus (SpliMNPV). Sci Rep. 2016; 6: 1–9. doi: 10.1038/s41598-016-0001-8 27650818 PMC5030640

[45] Liu BH. Statistical genomics: linkage, mapping, and QTL analysis. CRC press. 2017.

[46] Botstein D, White RL, Skolnick M, Davis RW. Construction of a genetic linkage map in man using restriction fragment length polymorphisms. Am J Hum Gent. 1980; 32: 314. 6247908 PMC1686077

[47] Powell W, Morgante M, Andre C, Hanafey M, Vogel J, Tingey S, et al. The comparison of RFLP, RAPD, AFLP and SSR (microsatellite) markers for germplasm analysis. Mol Breed. 1996; 2: 225–38.

[48] Tessier C, David J, This P, Boursiquot JM, Charrier A. Optimization of the choice of molecular markers for varietal identification in Vitis vinifera L. Theor Appl Genet. 1999; 98: 171–177.

[49] Prevost A, Wilkinson MJ. A new system of comparing PCR primers applied to ISSR fingerprinting of potato cultivars. Theor Appl Genet 1999; 98: 107–12.

[50] Rodriguez-Saona L, Giusti MM, Shotts M. Advances in infrared spectroscopy for food authenticity testing. Advances in Food Authenticity Testing, 2016; 71–116.

[51] Gopal M, Kumar R, Goswami A. Nano-pesticides—A recent approach for pest control. J Plant Prot Sci. 2012; 4: 1–7.

[52] Insecticide Resistance Action Committee. IRAC Mode of Action Classification Scheme. IRAC. 2020; v 9.4.

[53] Cordova D, Benner EA, Sacher MD, Rauh JJ, Sopa JS, Lahm GP, et al. Anthranilicdiamides: A new class of insecticides with a novel mode of action, ryanodine receptor activation. Pestic Biochem Physiol. 2006; 84: 196–214A.

[54] Lihl C, Heckel B, Grzybkowska A, Dybala-Defratyka A, Ponsin V, Torrentó C, et al. Compound-specific chlorine isotope fractionation in biodegradation of atrazine. Environmental Science: Processes & Impacts. 2020; 3: 792–801. doi: 10.1039/c9em00503j 32091522

[55] Camargo JA. (1991). Toxic effects of residual chlorine on larvae of *Hydropsyche pellucidula* (Trichoptera, Hydropsychidae): A proposal of biological indicator. Bull Environ Contam Toxicol. 1991; 47: 261–265. doi: 10.1007/BF01688649 1912702

[56] Institute of Medicine of the National Academies. Chapter 7: Dietary Reference Intakes for Water, Potassium, Sodium, Chloride, and Sulfate; National Academies Press: Washington, DC. 2005.

[57] UConn Home and Garden Education Center. (http://www.ladybug.uconn.edu/FactSheets/insecticides—low-toxicity-options.php). 2017.

[58] Radwan EM, Taha HS. Efficacy of certain pesticides against larvae of Tomato Leafminer, *Tuta absoluta* (Meyrick) (Lepidoptera: Gelechiidae). Egypt Acad J Boil Sci. 2017; 9: 81–95.

[59] Hosseinzadeh A, Aramideh S, Ghassemi-Kahrizeh A. Efficacy of bio-insecticides on *Tuta absoluta* (Meyrick) (Lep.: Gelechiidae) in laboratory and field conditions. Agric Eng Int. 2019; 21: 164–170.

[60] Lai T, Su JY. Effects of chlorantraniliprole on development and reproduction of beet armyworm, *Spodoptera exigua* (Hübner). J Pest Sci 2011; 84: 381–386.

[61] Cao GC, Lu Q, Zhang L, Guo F, Liang G, Wu K, et al. Toxicity of chlorantraniliprole to Cry1Ac-susceptible and resistant strains of *Helicoverpa armigera*. Pestic Biochem Physiol. 2010; 98: 99–103.

[62] He F, Shiang S, Haili T, Xiao S, Chao Q, Shoumin J, et al. Chlorantraniliprole against the black cutworm *Agrotis ipsilon* (Lepidoptera: Noctuidae): from biochemical/ physiological to demographic responses. Sci Rep. 2019; 9: 1–17. doi: 10.1038/s41598-018-37186-2 31316142 PMC6637144

[63] Yin X-H, Wu Q-J, Li X-F, Zhang Y-J, Xu B-Y. Sublethal effects of spinosad on *Plutella xylostella* (Lepidoptera: Yponomeutidae). Crop Prot. 2008; 27: 1385–1391.

[64] Wang P, Zhou L-L, Yang F, Li M, Liu X-M, Wang Y, et al. Sublethal effects of thiamethoxam on the demographic parameters of *Myzus persicae* (Hemiptera: Aphididae). J Econ Entomol. 2017; 110: 1750–1754. doi: 10.1093/jee/tox112 28520891

[65] Lutz AL, Bertokaccini I, Scotta RR, Curis MC, Favaro MA, Fernandez LN, et al. Lethal and sublethal effects of chlorantraniliprole on *Spodoptera cosmioides* (Lepidoptera: Noctuidae). Pest Manag Sci. 2018; 74: 2817–2821. doi: 10.1002/ps.5070 29766638

[66] El-Dewy MEH. Influence of some novel insecticides on physiological and biological aspects of *Spodoptera littoralis* (Boisduval). Alex Sci Exchange J. 2017; 38: 250–258.

[67] Han WS, Zhang S, Shen F, Liu M, Ren C, Gao X. Residual toxicity and sublethal effects of chlorantraniliprole on *Plutella xylostella* (Lepidoptera: Plutellidae). Pest Manag Sci. 2012; 68: 1184–1190. doi: 10.1002/ps.3282 22492544

[68] Banerjee BD, Seth V, Ahmed RS. Pesticide-induced oxidative stress: perspectives and trends. Rev Environ Health. 2001; 16: 1–36. doi: 10.1515/reveh.2001.16.1.1 11354540

[69] Afolabi OK, Aderibigbe FA, Folarin DT, Arinola A, Wusu AD. Oxidative stress and inflammation following sub-lethal oral exposure of cypermethrin in rats: Mitigating potential of epicatechin. Heliyon. 2019; 5: 125–134. doi: 10.1016/j.heliyon.2019.e02274 31440603 PMC6700339

[70] Bednářová A, Kodrík D, Krishnan N. Adipokinetic hormone exerts its anti-oxidative effects using a conserved signal-transduction mechanism involving both PKC and cAMP by mobilizing extra- and intracellular Ca2+ stores. Comp Biochem Physiol. 2013; 158: 142–149. doi: 10.1016/j.cbpc.2013.07.002 23845878

[71] Valavanidis T, Vlahogianni M, Dassenakis M. Scoullos Molecular biomarkers of oxidative stress in aquatic organisms in relation to toxic environmental pollutants Ecotoxicol Environ Saf. 2006; 64: 178–189. doi: 10.1016/j.ecoenv.2005.03.013 16406578

[72] Draper HH, Squires EJ, Mahmooch H, Wu S, Agarwal M, Handley A. Comparative evaluation of thiobarbituric acid methods for the determination of malondialdehydein biological materials. Free Radic Biol Med. 1993; 15: 353–363. doi: 10.1016/0891-5849(93)90035-s 8225017

[73] Wu GC, Bornman JE, Bennett SJ, Clarke MW, Fang ZX, Johnson SK. Individual polyphenolic profiles and antioxidant activity in sorghum grains are influenced by very low and high solar UV radiation and genotype. J Cereal Sci. 2017; 77: 17–23.

[74] Zhang QM, Zhu LS, Wang J, Xie H, Wang JH, Han YN, et al. Oxidative stress and lipid peroxidation in the earthworm *Eiseniafetida* induced by low doses of fomesafen. Environ Sci Pollut Res. 2013; 20: 201–208. 10.1007/s11356-012-0962-5 22585392

[75] Loewe L, Hill WG. The population genetics of mutations: good, bad and in different. Philos T R Soc B. 2010; 365: 1153–1167. 10.1098/rstb.2009.0317 PMC2871823 20308090

[76] Michod RE. Eros and Evolution. A Natural Philosophy of Sex, Addison-Wesley Publishing, USA.1995.

